# Pulmonary Vein Isolation Lesion Set Assessment During Radiofrequency Catheter Ablation for Atrial Fibrillation

**DOI:** 10.19102/icrm.2017.080204

**Published:** 2017-02-15

**Authors:** Edward Sze, Tristram D. Bahnson

**Affiliations:** ^1^Duke Center for Atrial Fibrillation and the Clinical Cardiac Electrophysiology Section, Duke University and Duke University Medical Center, Durham, NC

**Keywords:** Atrial fibrillation, catheter ablation, lesion set assessment, pulmonary vein isolation

## Abstract

This article reviews methods for lesion set assessment during radiofrequency catheter ablation for atrial fibrillation (AF). Pulmonary vein isolation (PVI) is the foundation for AF ablation, but PV reconnection can lead to treatment failure. Testing for entrance block can help confirm PVI, although complex electrograms that consist of both near- and far-field potentials may make assessment of entrance block challenging. Differential pacing maneuvers can help appropriately identify PV potentials. After entrance block has been achieved, pacing within the PVs to demonstrate capture of PV musculature with exit block may also help to confirm completeness of lesion sets for PVI. Employing a waiting period of at least 30 min or administering adenosine or isoproterenol can reveal dormant conduction, warranting adjunctive ablation. Additional techniques to confirm durable PVI include testing the ablation lines for excitability with high amplitude pacing, and automated waveform analysis of local electrogram morphology. Newer techniques like real-time magnetic resonance imaging and acoustic radiation force impulse elastography may have a role in testing the completeness of lesion sets in the future.

## Introduction

Left atrial (LA) radiofrequency catheter ablation (RFA) is a commonly performed treatment for atrial fibrillation (AF) among patients with significant symptoms or difficult-to-control ventricular rates.^1^ Prior research has shown that pulmonary vein (PV) musculature is arrhythmogenic, and commonly initiates AF.^2,3^ Consequently, the primary goal of most initial AF ablation procedures is to create circumferential lines of block at or near the PV antra to achieve permanent electrical isolation of the PV musculature from the atria.^4–9^ However, this isolation of the pulmonary vein (PVI) can be difficult to achieve, and requires the delivery of contiguous, transmural, and durable lesions (i.e., complete lesion sets). Despite successful acute PVI, a common cause of treatment failure appears to be late PV reconnection conduction recovery across ablation lines.^10,11^ Histology studies of patients with late PV reconnection have revealed that ablation lines are often non-contiguous and non-transmural, despite documentation of PVI during the index procedure.^12^

There have been many developments to enhance lesion delivery for the purpose of increasing the likelihood that complete lesion sets are delivered. The use of force-sensing catheters has improved procedural safety and facilitated more consistent RFA lesion delivery, and irrigated catheters allow for greater lesion sizes to be created without the need for increased temperatures at the tip-tissue interface, which may reduce the risk of thromboembolism.^9,13,14^ Electroanatomic mapping systems have evolved to help identify ablation targets and annotate where lesions are delivered, but still provide no direct information about actual lesion formation. Predictive algorithms like the force–time integral and VisiTag™ (Biosense Webster, Inc., Diamond Bar, CA, USA) have been incorporated into contemporary mapping systems in order to facilitate the delivery of complete lesions, but likewise do not provide direct assessments of lesion characteristics.^15,16^ Consequently, intraprocedural lesion assessment to confirm that complete RFA lesions have formed remains an important component of therapeutic catheter ablation for AF, and is the subject of this review.

### Assessing pulmonary vein entrance and exit block

**Figure 1:**
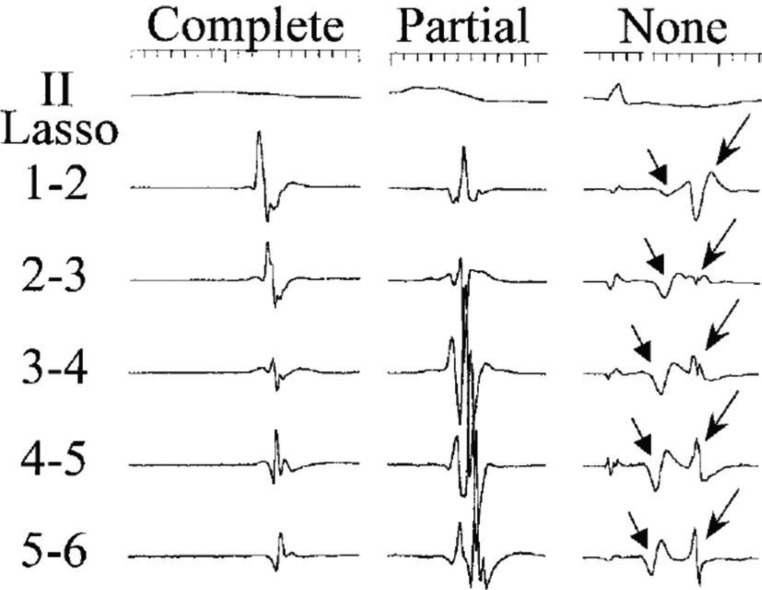
Recording from a circular mapping catheter showing overlap of far-field and near-field signals from the left atrium and pulmonary vein musculature, respectively. Adapted from: Tada H, Oral H, Greenstein R, Pelosi F Jr., Knight BP, Strickberger SA, Morady F. Differentiation of atrial and pulmonary vein potentials recorded circumferentially within pulmonary veins. *J Cardiovasc Electrophysiol.* 2002;13(2):118–123.

**Figure 2:**
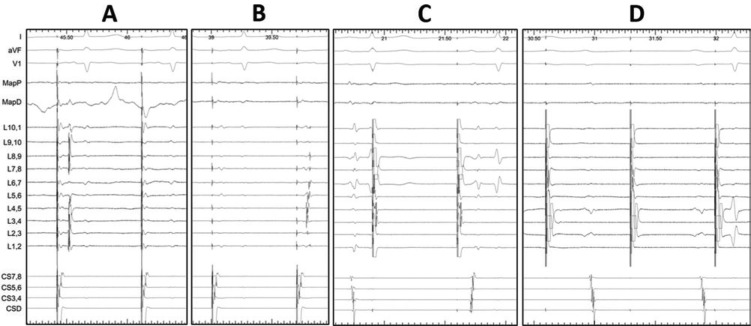
Circular mapping catheter recordings from within the pulmonary veins (PVs). (a) Recording from left inferior PV while pacing from the distal coronary sinus (CS) and performing ablation. Entrance conduction is evident on the first beat but disappears by the second beat, suggesting acute PV isolation. (b) Recording from the left superior PV while pacing distal CS. Adenosine bolus reveals dormant PV reconnection consistent with incomplete ablation near lasso pole 3. (c) Pacing of the left superior PV causes an evoked response fused with the pacing artifact on lasso poles 3/4 and 4/5. Exit conduction is evident after the second paced beat. (d) PV isolation with exit block despite adenosine bolus. Pacing from the lasso catheter induces evoked responses on poles 6/7 and 7/8. Note atrioventricular block because of adenosine but continued dissociation between the evoked responses and CS atrial activation.

The first described and most commonly used method to assess whether PVI lesion sets are complete is the documentation of conduction block into the targeted PVs. An important development in this regard was the invention of the circular mapping catheter, also called the circumferential mapping or “lasso” catheter, by Haissaguerre and colleagues.^17^ This deflectable decapolar or duo-decapolar catheter, with fixed or variable recording spline diameter, was designed for placement inside each ovoid PV to record electrograms (EGMs) and facilitate rapid identification and localization of electrical activity at the vein ostium or just within the PV. The lasso catheter continues to play a key role in lesion assessment today by facilitating the recognition of tissue conduction properties at ablated sites in order to achieve PVI. In sinus rhythm, a lasso catheter placed in a targeted PV will display a typical EGM in which the far-field LA signal occurs first, and is followed by an isoelectric interval that reflects the delay in impulse propagation across the PV ostia region, which is then followed by sharp deflections that represent activation of the near-field PV musculature **(Figure 1)**.^18^ When entrance block has occurred, near-field PV signals at all lasso poles will disappear **(Figure 2a)**.

Interpretation of endocardial EGMs recorded by the circular mapping catheter can be complex and challenging. Endocardial EGMs represent not only near-field signals from the PV musculature but also far-field signals recorded from the left ventricle, left atrial appendage and/or adjacent left atrium for left PVs, and the right atrium and/or superior vena cava (SVC) for right pulmonary veins (RPVs).^19–21^ Not all PVs display a typical activation pattern, as far-field signals may be absent, or distinct EGMs can overlap and merge to appear as one complex EGM **(Figure 1)**.^18^ Furthermore, ablation around the ostia of the PVs can alter near-field PV musculature signals, making the near-field EGM morphology similar to that of far-field signals.^19^ In one study of patients undergoing PVI, only 65% of patients had distinct atrial and PV signals when the right superior PV was interrogated in sinus rhythm, and only 3% of patients had distinct atrial and PV signals in left PVs.^18^

**Figure 3:**
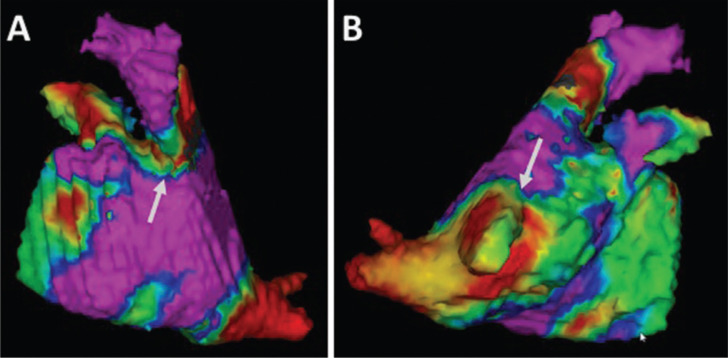
Color projection of a voltage map onto a segmented and registered magnetic resonance imaging of the left atrium. This map represents endocardial peak-to-peak bipolar voltage (color scale from 0.3–1.5 mV, red-yellow-green-blue-purple) created with a high density 20 pole-mapping catheter (PentaRay^®^, Biosense Webster, Inc., Diamond Bar, CA, USA). It reveals gaps of incomplete ablation (arrows). Low voltage signals in the superior portion of the right inferior pulmonary vein in this image were determined to be far field.

Pacing maneuvers are valuable for the purpose of distinguishing near- and far-field EGMs by allowing for the observation of local EGM timing in response to pacing from a variety of endocardial sites or at different rates.^18,19^ PV potentials generally exhibit decremental properties whereby high rate pacing may increase conduction delay into the PV, causing the putative PV signal to be “pushed out” or delayed relative to baseline, confirming a near-field PV signal.^19^ Unfortunately, manifest decremental conduction into the PV is not always present. A second strategy to distinguish LA from local PV EGMs is to pace from a variety of atrial sites and observe changes in the relative timing of the local EGM components (i.e., differential pacing). Pacing the left atrial appendage or distal coronary sinus (CS) when interrogating LPVs and pacing the high right atrium or SVC when interrogating the RPVs, can help to distinguish near- from far-field signals recorded with the lasso catheter.^20–22^ Specifically, the activation times between pacing artifact and far-field atrial potentials is shorter than for true residual PV potentials. When CS pacing fails to produce clear separation between local signal components, pacing even closer to the interrogated PV may be helpful (i.e., in the LA appendage or just outside of the left PVs; and within the high RA, SVC, or just outside the PV ostia for right PVs). The use of other multipolar mapping catheters like the PentaRay^®^ NAV catheter (Biosense Webster, Inc., Diamond Bar, CA, USA) or a mapping catheter in combination with annotation of voltage, or activation using an electroanatomic mapping system, can also be used to assess for entry block and PVI **(Figure 3)**.^23,24^

Initial reports have focused on the documentation of entry block for confirmation of PVI.^3^ Subsequent studies, however, have suggested that the pulmonary vein muscle sleeve could be paced, the local evoked responses observed and the presence or absence of exit block confirmed as well **(Figure 2)**.^25,26^ Although entry and exit block are often associated, cases have been reported in which exit conduction is demonstrable despite evidence of entry block.^25,26^ The demonstration of exit block during confirmed local capture of the PV also confirms the origin of putative far-field potentials because there will be dissociation between paced evoked responses of the PV musculature and far-field LA potentials **(Figure 2d)**. A previous study on exit conduction, completed by pacing sequentially around the lasso bipoles when positioned within a PV, suggested that this method might be superior to documentation of entry block because 28 out of the 66 PVs demonstrating entry block had evidence for exit conduction.^25^

One explanation for why bidirectional block may not occur with an initial ablation line may relate to anisotropy of wave-fronts approaching the PVs, which may not allow for conduction into the veins, although exit conduction is still intact. A second factor favoring exit conduction assessment is that this method allows for a clear distinction to be made between near- and far-field signals that become dissociated when exit block occurs. Further, exit conduction is easily recognized, if existent, because of capture of the atrial rhythm. Discrepancies between entry and exit block may be dependent in part on the ablation technique used, as a two-center study of 378 subjects did not find a significant dissociation between the occurrence of entry versus exit block during assessment for PVI, and reported only 0.6% of PVs demonstrated unidirectional block.^26^ Far-field capture of adjacent atrial tissue during pacing to assess for exit block can also complicate this endpoint assessment by mimicking intact exit conduction; this should be considered as a possibility when exit conduction is evident.^27^ In particular, high output pacing from within the left superior pulmonary vein can frequently capture the LA appendage, and pacing within the right PVs can capture the superior vena cava.^27,28^

In our laboratory, sequentially pacing from all bipoles of the circular mapping catheter with stimulus strengths of 3 V to 5 V at 2 ms pulse-width, or with a minimum output to reveal local evoked responses that prove PV capture, is employed to minimize the likelihood of far-field atrial capture during pacing from within the PVs **(Figure 2)**.

Other observations that have bearing on the assessment of the completeness of RFA lesion sets have to do with the excitability properties of the PV musculature. Loss of the ability to capture the PV musculature during pacing within the pulmonary veins has been associated with acute durable entry block and adenosine resistance, suggesting that this finding is equivalent to documented exit block.^29^ Another study suggested that identification of robust dissociated PV potentials following achievement of acute PVI may be associated with incomplete ablation and PVs destined to reconnect during a 30-min waiting period.^30^

### Assessing for dormant PV connection

High recurrence rates of AF after ablation, together with high rates of documented PV reconnection during repeat procedures, suggest that confirmation of acute entry and exit block is necessary but insufficient to confirm that the delivered lesion sets (comprised of permanent, contiguous and transmural lesions) are complete. Two observations underscore this hypothesis: first, intraprocedural PV reconnection following acute documentation of entry and exit block frequently occurs following a waiting period; and, second, adenosine or isoproterenol infusion has been reported to reveal transient or dormant PV reconnection.^31,32^ We will consider these observations in more detail.

Prior studies have examined the use of a waiting period after documentation of acute PVI as a method to reveal regions of incomplete ablation. One study compared 30- and 60-min waiting periods and reported that more PV reconnections were seen with the longer waiting time, suggesting that even a 60-min delay before assessing for PVI may be insufficient to identify all sites of incomplete ablation that are destined to cause PV reconnection.^31^

Unsurprisingly, a prospective randomized study of waiting periods of at least 60 min accompanied by a strategy to ablate all sites of PV reconnection failed to show any improvement in AF recurrence rates, further suggesting that this approach may be of limited value.^33^

Adenosine has been shown to activate outward potassium currents in cardiac myocytes and hyperpolarize membrane potentials, which in turn shortens the cardiac action potential and improves the safety factor for impulse propagation through recruitment of sodium channels.^34^ Arentz et al.^32^ reported that adenosine bolus infusion of 12–18 mg resulted in transient PV reconnection in 25% of pulmonary veins targeted with RFA for which entry block had been acutely documented. Subsequent studies have confirmed this finding of an adenosine-sensitive “dormant” PV connection in up to 35% of targeted PVs that demonstrate acute entry block. Cheung et al.^35^ reported on the timing of adenosine-induced PV reconnection and found it to be concurrent with AV block, further supporting the notion that the drug reveals dormant PV connection by activating the acetylcholine-sensitive potassium channel (I_K_(_ACh_)), hyperpolarizing or repolarizing injured (depolarized) myocytes, and thus potentiating conduction across the injured regions. In addition, this study also showed that adenosine-sensitive dormant PV connections were only seen after the initial dromotropic effects of adenosine on AV node function became evident, and would never first occur *after* the dromotropic effects of the adenosine had resolved (although dormant PV connection could persist after dromotropic effects resolved). This suggests that the effect was not due to reflex vagal withdrawal or changed sympathetic tone.^35^ The dosing of adenosine was considered by Kapa et al., who suggested that persistent PVI after a dose sufficient to produce AV block was minimally required to exclude dormant PV reconnection.^36^ Interestingly, concurrent use of dipyridamole has been reported to prolong the effects of adenosine on the order to minutes, and has been used to facilitate activation mapping to guide adjunctive RFA when accurate localization of target sites was otherwise hindered by the transient nature of dormant reconnection, although ventricular pacing support was required.^37^

Previous reports have also suggested that isoproterenol infusion can reveal dormant PV reconnection. To compare adenosine with isoproterenol, Datino et al. reported on 25 subjects undergoing PVI and assessed the occurrence of dormant conduction with sequential use of adenosine and isoproterenol, and a combination of adenosine and isoproterenol.^38^ This study confirmed both a high incidence of dormant PV connection in human subjects (i.e., 31% of veins) and also demonstrated that the use of isoproterenol alone (up to 20 mg/min for >50% heart rate increase) revealed only 10% of reconnection sites, whereas adenosine (6 mg or more to produce at least one instance of AV block) revealed 87% of reconnection sites.^38^ In this study, the addition of isoproterenol to adenosine revealed an additional 13% of observed reconnection sites. Furthermore, the investigators measured cellular electrical potential in the region of lesion delivery after PVI in canines and demonstrated a hyperpolarizing effect of 3.8 mV±0.6 mV with isoproterenol versus 9.1 mV±0.6 mV for adenosine, with no additive change in membrane potential with both medications given together.^38^ Accordingly, there is some doubt whether adding isoproterenol to adenosine significantly increases the recognition of dormant PV reconnection.

Other studies have compared adjunctive adenosine infusion to the use of a waiting period after achieving PVI. In one study, dormant PV reconnection was reported in 64% of 88 subjects. Although 94% of the reconnection sites in this study became manifest during a 30-min waiting period, 6% did not, suggesting that adenosine infusion was superior to waiting for 30 min to identify regions of incomplete ablation.^39^ Consistent with these findings, another study published in the same year reported that 25% of PVs reconnected during the waiting period, and an additional 12% demonstrated dormant PV reconnection after a 30-mg bolus adenosine infusion at the end of the waiting period.^40^

The use of adenosine to reveal dormant PV reconnection during assessment of lesion set completeness has also been reported to improve outcomes after RFA for AF. An initial case–control study of adenosine-resistant PV isolation as a lesion assessment endpoint found that dormant PV reconnection was seen in 73% of subjects who received adenosine, and that adjunctive ablation to eliminate dormant PV reconnection improved AF rhythm controls at 6 months.^41^ A second larger case–control study involving 148 subjects also reported improvement in AF rhythm control using adenosine-guided (20 mg adenosine triphosphate during isoproterenol infusion) ablation of dormant PV reconnection.^42^ Interestingly, the use of adenosine to confirm RFA lesion set completeness has also been reported for superior vena cava isolation and cavotricuspid isthmus ablation.^43–45^

### Strategies for lesion assessment

Given the limitations of EGM analysis alone for the assessment of entry and exit block to confirm that the lesion sets are complete, additional techniques have been reported.^46^ One such method involves pacing at high output using the mapping ablation catheter along the ablation line and targeting areas of capture with adjunctive ablation. This technique, known as “pace-capture,” uses documented non-excitability throughout the ablation regions as the lesion set assessment endpoint.^47^ A subsequent prospective randomized study completed by Steven et al. compared bidirectional block versus bidirectional block plus documented non-excitability along the ablation line as the procedure endpoint. They found that although procedure duration for the group with pace-capture assessment was on average 46 min longer, there was a significant reduction in AF during post-procedure monitoring in this group.^48^ Non-excitability was also associated with a lower amplitude signal along the ablation line, although the presence of low amplitude signals alone was not predictive. These findings suggest that pace-capture as a lesion assessment endpoint may improve some outcomes. Finally, the examination of EGM morphology has been proposed as a method for achieving and confirming PVI.^49,50^ In particular, waveform analysis of local EGM morphology may allow for the automated confirmation of PVI status, though this method is not currently used widely.^50^

### Investigational strategies for lesion assessment

Existing methods for the performance of lesion set assessment are indirect. The use of endocardial EGM-based assessment for PV entry and exit block only provides information on the acute excitability characteristics of the target regions, and inexcitability can occur in some cases because of reversible tissue injury. Such sites likely produce PV reconnection in the future. Factors such as possible RFA-induced tissue edema, loss of excitability because of cellular injury with depolarization and inactivation of sodium channels, or ablation-induced increases in tissue anisotrophy are factors that would be expected to dissociate acute tissue excitability characteristics from long-term PVI status. Likewise, pace-capture techniques also rely on tissue excitability to confirm achievement of complete ablation.

Magnetic resonance imaging (MRI) of the heart is a powerful imaging technique that provides direct information on the presence or absence of viable tissue, and tissue edema. Previous studies have suggested that MRI scans can identify ablated regions in the atrium, and can also identify regions of complete ablation.^51^ Canine studies have further reported that MRI can visualize gaps in lesion sets that correlate with sites of PV reconnection and with histologic incomplete ablation.^52^ In addition, proof-of-concept studies describe the use of intraprocedural MRI to guide ablation to identify and close gaps in incomplete ablation lines in swine, and described the use of specialized catheters with the ability to record endocardial EGMs and deliver ablative RF energy under MRI guidance.^53,54^ Although promising, real-time or near real-time intraprocedural MRI guidance of RFA lesion delivery to achieve durable PVI has its restrictions, including a lack of widely available catheters that are MRI compliant, lengthy image acquisition times, a limited ability to resolve small discrete RFA lesions, and the high levels of cost and skill required to integrate MRI equipment into or adjacent to the clinical electrophysiology laboratory.

**Figure 4:**
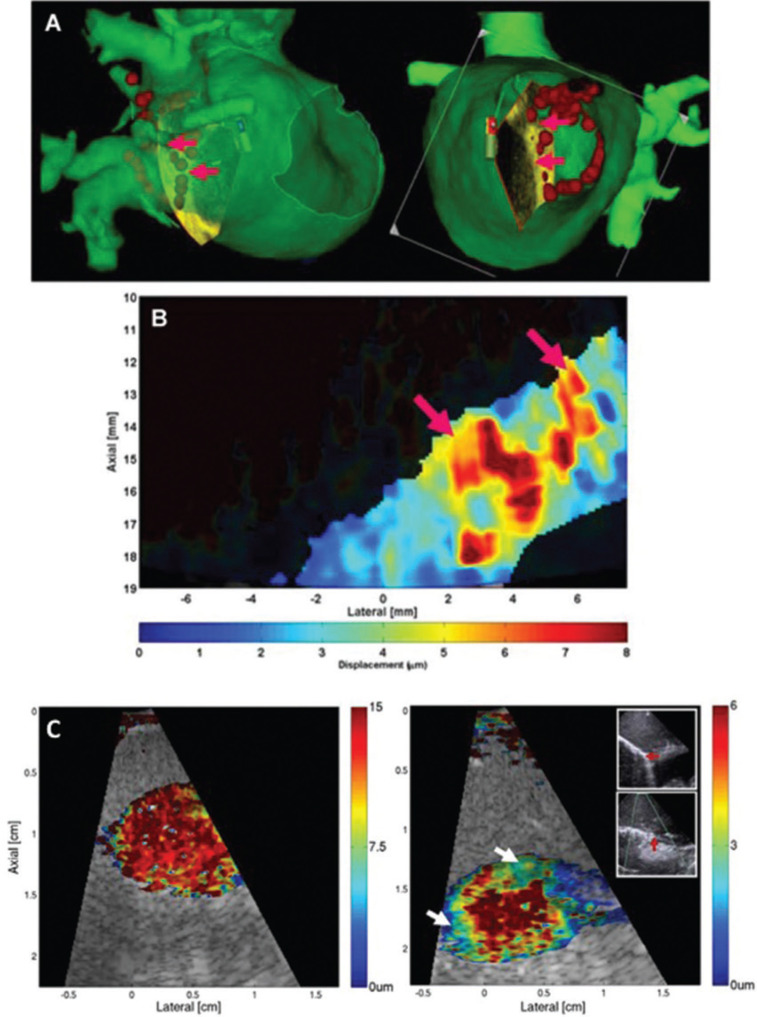
Acoustic radiation force impulse elastography (ARFIE) reveals regions of incomplete ablation. (a) Two views of a segmented and registered computed tomography scan. The intracardiac echocardiography imaging plane is seen projected on the sepal margin of the right inferior pulmonary vein (PV) antrum and yields the image in (b). Radiofrequency ablation (RFA) tags are marked in red, and the red arrows designate regions with intact conduction (noted on lasso recordings). (b) The ARFIE image demonstrating stiff ablated areas (blue) and soft un-ablated areas (red) corresponding with sites of incomplete ablation and PV connection. (c) A left atrial ARFIE image of the ligament of Marshall before ablation; the tissue was uniformly soft (appearing red) consistent with unablated tissue (left). After two RF pulses (see inset), the ARFIE image shows two discrete lesions that are incomplete (arrows). Adapted from: Bahnson TD, Eyerly SA, Hollender PJ, et al. Feasibility of near real-time lesion assessment during radiofrequency catheter ablation in humans using acoustic radiation force impulse imaging. *J Cardiovasc Electrophysiol.* 2014;25(12):1275–1283.

A new ultrasound imaging technique has been developed for the purpose of obtaining information about tissue elasticity or stiffness. Known as acoustic radiation force impulse elastography (ARFIE), current iterations of the technique generate pseudo-colored images that are superimposed onto a two-dimensional ultrasound image to help users visualize local tissue elasticity properties that correspond with each image pixel within the field of view.^55^ When cardiac tissue is ablated with RF energy, heat-induced coagulation occurs, producing stiff regions with well-demarcated boundaries that correspond to the extent of the RFA lesion in muscle preparations.^56^ These regions are less elastic (i.e., more stiff) than the adjacent un-ablated myocardium, enabling ARFIE imaging to visualize the RFA lesions *in situ.* Intracardiac echocardiography (ICE) is routinely used during complex ablation procedures including RFA for AF, and ARFIE imaging has been adapted to work with clinical electrophysiology imaging systems.^57,58^ In animals, gaps in the ablation line corresponding with activation mapping-determined conduction, and regions with complete ablation corresponding with documented conduction block, have been visualized with ARFIE.^58^ Preliminary research has demonstrated that RFA lesions can also be identified during clinical RFA procedures, and that lesion formation, including the size and extent of the RFA lesions, can be evaluated in near real time (i.e., seconds).^57,59^ Furthermore, regions of incomplete ablation with intact conduction have also been resolved using this system during clinical RFA for AF and typical atrial flutter **(Figure 4)**.^57,59^*In vitro* imaging has further demonstrated the capacity of this system to perform near real-time monitoring of lesion formation.^60^ In this study, visualization of the evolving RFA lesion was reported. Animal studies have revealed that the extent of RFA lesions are stable over time, further suggesting that this imaging method may be able to distinguish durable lesions from regions with incomplete ablation due to reversibly injured myocytes injured cells or associated tissue edema.^61^ It is not yet known whether this imaging method is capable of identifying regions with incomplete ablation that demonstrate dormant conduction or are otherwise-destined to produce PV reconnection in the future.

Both MRI and ARFIE provide direct information about the presence and extent of RFA lesions delivered in the atria, and hold significant promise to provide additional useful information to help clinicians distinguish injured but incompletely ablated cardiac tissue from completely ablated tissue that is not expected to recover in the future and contribute to treatment failure.

## Conclusions

The confirmation of complete lesion sets is an integral part of therapeutic ablation for AF, including PVI. Given the high incidence of PV reconnection and arrhythmia recurrence following RFA for AF, it remains important to carefully evaluate the completeness of delivered lesion sets by confirming entry and exit block, using adenosine to reveal dormant PV connections, and applying pacecapture techniques as an adjunct to EGM interpretation, when needed. Current lesion assessment methods are indirect and often insensitive for the purpose of distinguishing injured tissue that is destined to produce PV reconnection in the future from completely ablated tissue. New imaging methods to reveal tissue characteristics, apart from functional properties, hold promise to improve lesion assessment and outcomes.

## Figures and Tables

**Table 1:** Lesion Set Assessment to Confirm Pulmonary Vein Isolation

**Indirect methods** Documentation of entry block with circular mapping catheter (lasso) Documentation of entrance block with electroanatomic mapping (voltage or activation) Documentation of exit block Confirming non-excitability during pace-capture mapping at lesion sites Confirming no dormant pulmonary vein reconnection (entry- or exit block) with adenosine (± isoproterenol) **Direct methods (investigational)** Magnetic resonance imaging for Incomplete Lesions or Gaps Acousitic radiation force impulse elastography imaging of gaps or incomplete radiofrequency ablation
